# Protective Effect of *TNFRSF11A rs7239667 G* > *C* Gene Polymorphism on Coronary Outcome of Kawasaki Disease in Southern Chinese Population

**DOI:** 10.3389/fgene.2021.691282

**Published:** 2021-08-17

**Authors:** Linyuan Zhang, Kun Lin, Yishuai Wang, Hongyan Yu, Jinqing Li, Lanyan Fu, Yufen Xu, Bing Wei, Hanran Mai, Zhiyong Jiang, Di Che, Lei Pi, Xiaoqiong Gu

**Affiliations:** ^1^Department of Clinical Biological Resource Bank, Guangzhou Institute of Pediatrics, Guangzhou Women and Children’s Medical Center, Guangzhou Medical University, Guangzhou, China; ^2^Department of Blood Transfusion and Clinical Laboratory, Guangzhou Institute of Pediatrics, Guangzhou Women and Children’s Medical Center, Guangzhou Medical University, Guangzhou, China; ^3^School of Medicine, South China University of Technology, Guangzhou, China

**Keywords:** Kawasaki disease (KD), coronary artery lesion (CAL), tumor necrosis factor receptor superfamily, single nucleotide polymorphisms (SNP), tumor necrosis factor superfamily

## Abstract

**Background:**

The main symptoms of Kawasaki disease (KD) are inflammatory vasculitis characterized by fever lasting 1–2 weeks, failure to respond to antibiotic treatment, conjunctivitis, redness of the lips and mouth, strawberry tongue, and painless enlargement of the neck lymph nodes. Studies have been shown that tumor necrosis factor (TNF) and TNF receptor family members are abnormally expressed in the acute phase of Kawasaki disease, also revealing that these two play a significant role in the pathogenesis of KD. The purpose of our study is to determine the relationship between *TNFRSF11A rs7239667* and the pathogenesis of KD and Coronary artery lesions in KD.

**Methods and Results:**

In this study, *TNFRSF11A* (*rs7239667*) genotyping was performed in 1396 patients with KD and 1673 healthy controls. Our results showed that *G* > *C* polymorphism of *TNFRSF11A* (*rs7239667*) was not associated with KD susceptibility. In addition, the patients with KD were divided into CAA and NCAA groups according to whether they had coronary artery aneurysm (CAA) or not, and the *TNFRSF11A rs7239667* genotyping was performed in the two groups. After gender and age calibration, We found that genotype CC of *TNFRSF11A* may be a protective factor in KD coronary artery damage (adjusted OR = 0.69 95% CI = 0.49–0.99 *P* = 0.0429) and is more significant in children with KD ≤ 60 months (adjusted OR = 0.49 95% CI = 0.49–0.93 *P* = 0.0173).

**Conclusion:**

Our study suggests that *TNFRSF11A rs7239667 G* > *C* polymorphism maybe play a protective gene role for the severity of KD coronary artery injury and is related to age, which has not been previously revealed.

## Introduction

Kawasaki disease is an immune angioinflammatory disease characterized by a fever that persists for 1–2 weeks, conjunctivitis, redness of the lips and mouth, enlarged non-suppurative lymph nodes in the neck, and peeling of the hands and feet (Kato et al., 1975; Newburger et al., 2004). Coronary artery disease is the most common and intractable complication of KD. In the most serious cases, it can become coronary artery aneurysm (CAA) and endanger the life of patients (Tacke et al., 2014). Coronary artery lesion (CAL) caused by KD have became the most common cause of acquired heart disease in children in some countries (Newburger et al., 2004; Tacke et al., 2014; Singh et al., 2015; Kumrah et al., 2020). Therefore, intravenous gamma globulin is often used for the anti-inflammatory treatment of acute phase of KD in clinic (Gupta et al., 2001; Kumrah et al., 2020). At present, it has been more than 50 years since the first diagnosed KD case, and researches on its etiology and pathogenesis continue continuously, but its etiology and pathogenesis are still an unsolved mystery (Tacke et al., 2012). A growing number of studies have claimed that genetic variations associated with immune response function are associated with increased susceptibility to KD and development of CAL (Kuo et al., 2015; Kumrah et al., 2020).

Tumor necrosis factor α(TNF-α), a member of Tumor necrosis factor superfamily (Aggarwal, 2003), is one of the cytokines produced by immune cells during inflammation (Gupta et al., 2001; Wang et al., 2011; Kumrah et al., 2020). It is believed that it plays an indispensable role in the body’s resistance to infection and immune response (Yamaji et al., 2019; Halim et al., 2021). Numerous reports have confirmed that high level of TNF can induce inflammatory response in children with KD, and is closely related to vascular endothelial damage and the incidence of CAA in KD closely (Fiers, 1991; Aeschlimann and Yeung, 2016). In addition, Furukawa et al. (1994) showed that TNF-TNF receptor signals were abnormally activated in the acute phase of KD. Yasumura et al. (2020) found that ratio of sTNFR-I/II was lower in both the acute and non-acute phases of TNF-recurrent syndrome than that in autoinflammatory diseases including KD Mizuta et al. (2021) showed that serum levels of STNFR-1, STNFR-2, and STNFR-I/II were significantly higher in patients with KD complicated with macrophage activation syndrome than that in patients with acute KD (Jinkawa et al., 2019). Stringer and Yeung (2008) suggested that TNFRs contained several functional motifs which were interacted with intracellular proteins, directed intracellular signal transduction, and further activated transcription factors, which ultimately led to increasing expression of pro-inflammatory cytokines and leukocyte recruitment. Weiss first reported that the TNFR blocker infliximab can be used in children with KD who were resistant to Immunoglobulin C (Weiss et al., 2004). Moreover, some studies have further verified in animal experiments that the mouse model without tumor necrosis factor or using tumor necrosis factor blocker were not easy to develop CAL (Oharaseki et al., 2013). While Chien et al. (2003) found no significant correlation between TNF-α promoter region gene polymorphism and susceptibility to KD or CAL in Taiwan population. In addition, *TNF* and TNFRs promoter region gene polymorphisms may be associated with the occurrence of a variety of tumors (Gupta et al., 2008). More and more studies, have led us to speculate about the association between polymorphisms in other TNFR loci and KD. As a member of the tumor necrosis factor receptor superfamily, *TNFRSF11A* is also known as nuclear factor-κB receptor activator (RANK) (Yang et al., 2004). The RANK/RANK ligand (RANKL)/osteoprotection axis (RRO axis) was first identified in the immune system and skeletal system (Anderson et al., 1997; Lacey et al., 1998). As we all know, KD is also an immune vasculitis disease (Che et al., 2018b). However, there haven’t any reports on the relationship between *TNFRSF11A* gene polymorphism and susceptibility to KD and CAL. In the present study, we aimed to explore the association between *TNFRSF11A* (*rs7239667*) gene polymorphism and genetic susceptibility to KD and CAL in southern Chinese population.

## Materials and Methods

### Study Subjects

To investigate the effect of *TNFRSF11A* (*rs7239667*) gene polymorphism on the severity of coronary complications associated with KD, we enrolled 1396 patients with KD diagnosed at Guangzhou Women and Children’s Medical Center. All of KD patients who diagnosed according to the criteria of American Heart Association were enrolled from Guangzhou Women and Children’s Medical Center between January 2014 and December 2019 (Newburger et al., 2004; McCrindle et al., 2017). In addition, 1673 healthy age- and sex- matched children who underwent physical examinations at the hospital were selected as controls with the informed consent of each control person’s guardian. Each control donated 2 ml of blood for genomic DNA extraction. This study was approved by Children’s Medical Center of Guangzhou Women and Women’s Affairs Commission (2014073009).

### DNA Extraction and SNPs Genotyping

We melted all the collected whole blood, and then ensured that each tube was 200 μl of whole blood, according to the DNA extraction kit manufacturer instructions (Tiangen, Beijing, China) for DNA extraction using DNA quality, finally will be extracted to save until later use DNA −80°C. As above, after A extraction kit (Tiangen, Beijing, China), TaqMan method was used to genotype *TNFRSF11A rs7239667* polymorphisms. The PCR mixture (total volume was 10 μg, including 2× multiplex PCR mixture + template DNA to be amplified and PCR primers) was added to the 384-well plate, and the related detection was performed by ABI-Q6 PCR instrument. The primers were purchased from Thermo Fisher Scientific reagent company. The specific steps can be referred to our previous literature (Che et al., 2018b).

### Statistical Analysis

In this study, the genotype frequency and demographic variable χ^2^ test of each SNP were compared between KD cases and healthy controls. The Hardy – Weinberg equilibrium (HWE) of the samples was calculated using the chi-square goodness of fit test. The association between *TNFRSF11A* (*rs7239667*) polymorphism and KD susceptibility and coronary artery disease severity was assessed by calculation of odds ratio (OR) and 95% confidence interval (CI). Univariate unconditional logistic regression analysis was taken. The adjusted ORS were calculated by multivariate analysis adjusted for gender. SAS software was used for all statistical analyses (version 9.1; SAS Institute), *P* < 0.05 was deemed statistically significant.

## Results

### Clinical Characteristics of the Study Population

The distribution of age and sex between KD patients and healthy control group are shown in Table 1. The mean age and age distribution range of KD patients and healthy control group was 25.85 months (±21.85, range 1–156 months) and 33.67 months (±24.09, range 1–168 months), respectively. There was no significant difference in age (*P* = 0.1437) and gender (*P* = 0.2230) between KD patients and healthy control group.

**TABLE 1:** Frequency distributions of selected features in Kawasaki disease and control groups.

**Variables**	**Cases (*N* = 1396)**		**Controls (*N* = 1673)**		***P*^a^**
	**No.**	**%**	**No.**	**%**	
Age range, month	1–156		1–168		
Mean ± SD	25.85 ± 21.85		33.67 ± 24.09		0.1437
≤60	1305	93.48	1541	92.11	
>60	91	6.52	132	7.89	
**Gender**					
Male	886	63.47	1026	61.33	0.2230
Female	510	36.53	647	38.67	
**Coronary artery outcomes**					
CAA	408	29.23			
NCAA	988	70.78			

*CAA, coronary artery aneurysm; NCAA, no coronary artery aneurysm. ^*a*^Two-sided χ^2^ test for distributions between KD patients cases and controls.*

### Correlations Between *TNFRSF11A* Polymorphisms With the Risk of KD and the Severity of Coronary Complications in KD Patients

The SNP genotype distribution of selected *TNFRSF11A rs7239667 G/C* and its correlation with KD risk are shown in Table 2. The genotype frequency of the samples conforms to Hardy–Weinberg law. Unfortunately, we did not observe any significant association between the risk of SNP and KD. Patients with KD were then divided into CAA group and NCAA group according to whether they had coronary aneurysm (CAA) or not, and *TNFRSF11A rs7239667* genotyping was performed in the two groups (Table 3). After further adjustment for gender and age, we found that genotype CC of *TNFRSF11A* may be a protective factor for KD coronary artery injury (adjusted OR = 0.69 95% CI = 0.49–0.99 *P* = 0.0429) (Table 4). It was more significant in KD patients ≤60 months (adjusted OR = 0.49 95% CI = 0.49–0.93 *P* = 0.0173).

**TABLE 2:** Genotype distribution of *TNFRSF11A rs7239667 G* > *C* polymorphism and KD susceptibility.

**Genotype**	**Cases (*N* = 1396)**	**Controls (*N* = 1673)**	***P*^a^**	**OR (95% CI)**	***P***	**Adjusted OR (95% CI)^b^**	***P*^b^**
***TNFRSF11A/rs7239667 G > C* (HWE = 0.1644)**							
GG	408 (29.23)	511 (30.54)	0.0664	1.000		1.000	
GC	717 (51.36)	793 (47.40)		1.13 (0.96–1.34)	0.1390	1.14 (0.97–1.35)	0.1197
CC	271 (19.41)	369 (22.06)		0.92 (0.75–1.13)	0.4215	0.94 (0.76–1.16)	0.5582
Dominant	988 (70.77)	1162 (69.06)	0.4273	1.07 (0.91–1.24)	0.4281	1.08 (0.92–1.26)	0.3491
Recessive	1125 (80.59)	1304 (77.94)	0.0722	0.85 (0.71–1.02)	0.0728	0.87 (0.72–1.04)	0.1128

*^*a*^Two-sided χ^2^ test for distributions between Kawasaki disease patients and controls. ^*b*^Adjusted for age and gender.*

**TABLE 3:** The frequency distribution of selected features in Kawasaki patients with and without CAA.

**Variables**	**CAA (*N* = 408)**		**NCAA (*N* = 908)**		***P*^a^**
	**No.**	**%**	**No.**	**%**	
Age range, month	1–151		1–166		
Mean ± SD	23.54 ± 23.12		26.80 ± 21.23		0.7378
≤60	380	93.14	925	93.62	
>60	28	6.86	63	6.38	
**Gender**					
Male	303	74.26	583	59.01	<0.0001
Female	105	25.74	405	40.99	

*CAA, coronary artery aneurysm; NCAA, no coronary artery aneurysm. ^*a*^Two-sided χ^2^ test for distributions between KD with and without CAA.*

**TABLE 4:** *TNFRSF11A rs7239667 G > C* polymorphism and genotype distribution in KD patients with different coronary outcomes.

**Genotype**	**CAA (*N* = 408)**	**NCAA (*N* = 988)**	***P*^a^**	**OR (95% CI)**	***P***	**Adjusted OR (95% CI)**	***P*^b^**
***TNFRSF11A/rs7239667 G > C***							
GG	126 (30.88)	282 (28.54)	**0.0490**	1.000		1.000	
GC	219 (53.68)	498 (50.40)		0.98 (0.76–1.28)	0.9058	0.99 (0.76–1.29)	0.9363
CC	63 (15.44)	208 (21.05)		0.68 (0.48–0.96)	**0.0302**	0.69 (0.49–0.99)	**0.0429**
Dominant	282 (69.12)	706 (71.46)	0.3835	0.89 (0.70–1.15)	0.3821	0.90 (0.70–1.16)	0.4304
Recessive	345 (84.56)	780 (78.95)	**0.0142**	0.69 (0.50–0.93)	**0.0164**	0.70 (0.51–0.95)	**0.0238**

*CAA, coronary artery aneurysm; NCAA, no coronary artery aneurysm. ^*a*^χ^2^ tests were used to determine differences in genotype distributions between KD patients with and without CAA. ^*b*^Adjusted for age and gender. Bold characters are all values with *P* value < 0.05, indicating that this value has statistical significance.*

### Stratified Analysis

Then we further explored the relationship between *TNFRSF11A* gene and the prevalence of KD and the degree of coronary artery damage in patients with KD considering age and gender (Tables 5, 6). In terms of age, since KD occurs more frequently in children aged less than 60 months (Chu et al., 2017), we conducted stratified analysis of age by taking ≤60 months as the limit (Aeschlimann and Yeung, 2016). We found that male patients with KD (OR = 0.79, 95% CI = 0.64–0.99, *P* = 0.0425, adjusted OR = 0.80, 95% CI = 0.63–1.00 *P* = 0.0533) and patients with KD with CAA (OR = 0.65, 95% CI = 0.48–0.86, *P* = 0.0033, adjusted OR = 0.65, 95% CI = 0.48–0.88, *P* = 0.045), the risk of developing KD in children with *rs7239667 CC* genotype was significantly lower than that in children with *GG/GC* genotype. In addition, children with rs7239667 *CC* genotype with KD are less likely to develop coronary aneurysms. However, no such correlation is found in Table 5.

**TABLE 5:** Stratified analysis of the association between *TNFRSF11A* polymorphism and the risk of KD patients with CAA in a population of southern China.

**Variables**	**GG/GC**	**CC**	***P*^a^**	**OR (95% CI)**	***P***	**Adjusted OR (95% CI)**	***P*^b^**
	**CAA/NCAA**						
**Age, months**							
≤60	323/730	57/195	**0.0115**	0.66 (0.48–0.91)	**0.0119**	0.67 (0.49–0.93)	**0.0173**
>60	22/50	6/13	0.9316	1.05 (0.35–3.12)	0.9311	1.05 (0.35–3.13)	0.9370
**Gender**							
Male	257/463	46/120	0.0506	0.69 (0.48–1.00)	0.0515	0.70 (0.48–1.01)	0.0576
Female	88/317	17/88	0.2011	0.70 (0.39–1.23)	0.2131	0.70 (0.39–1.23)	0.2136
**Coronary artery lesion**							
CAA	64/780	9/208	0.0594	0.53 (0.26–1.08)	0.0791	0.52 (0.25–1.07)	0.0775
CAL	77/780	16/208	0.3716	0.78 (0.45–1.36)	0.3826	0.79 (0.45–1.38)	0.4050
NCAL	204/780	38/208	0.0564	0.70 (0.48–1.02)	0.0634	0.71 (0.48–1.04)	0.0796

*CAA, coronary artery aneurysm; CAL, coronary artery lesion; NCAL, no coronary artery lesion; NCAA, no coronary artery aneurysm. ^*a*^χ^2^ tests were used to determine differences in genotype distributions between KD patients with and without CAA. ^*b*^Adjusted for age or/and gender. Bold characters are all values with *P* value < 0.05, indicating that this value has statistical significance.*

**TABLE 6:** Stratified analysis of the association between *TNFRSF11A* polymorphism and KD risk in southern China.

**Variables**	**GG/GC**	**CC**	***P*^a^**	**OR (95% CI)**	***P***	**Adjusted OR (95% CI)**	***P*^b^**
	**KD/controls**						
**Age, months**							
≤60	1053/1204	252/337	0.0927	0.86 (0.71–1.03)	0.0934	0.86 (0.71–1.03)	0.0953
>60	72/100	19/32	0.5553	0.83 (0.43–1.57)	0.5570	0.82 (0.42–1.60)	0.5559
**Gender**							
Male	720/795	166/231	**0.0418**	0.79 (0.64–0.99)	**0.0425**	0.80 (0.63–1.00)	0.0533
Female	405/509	105/138	0.7586	0.96 (0.72–1.27)	0.7591	0.97 (0.73–1.29)	0.8216
**KD subtypes**							
CAA	345/1304	63/369	**0.0024**	0.65 (0.48–0.86)	**0.0033**	0.65 (0.48–0.88)	**0.0045**
CAL	182/1304	43/369	0.3080	0.84 (0.59–1.19)	0.3150	0.86 (0.60–1.22)	0.3920
NCAL	598/1304	165/369	0.8113	0.98 (0.79–1.20)	0.8120	0.98 (0.80–1.21)	0.8598

*CAA, coronary artery aneurysm; CAL, coronary artery lesion; NCAL, no coronary artery lesion. ^*a*^Two-sided χ^2^ test for distributions between Kawasaki disease patients and controls. ^*b*^Adjusted for age or/and gender. Bold characters are all values with *P* value < 0.05, indicating that this value has statistical significance.*

## Discussion

In the present study, we evaluated the association between *TNFRSF11A* gene polymorphism (*rs7239667 C*) and susceptibility to KD and the severity of coronary artery damage in children with KD in 1396 patients (408 with CAA and 988 without CAA) and 1673 healthy controls. Our results showed that *TNFRSF11A* (*rs7239667*), the selected SNP, was not associated with KD susceptibility in children from southern China. Interestingly, when we compared KD patients with or without CAA, we found that *TNFRSF11A* (*rs7239667 C*) variant genotype significantly reduced the degree of coronary artery damage in KD patients.

Although the etiology and pathogenesis of KD remain unclear, systemic vascular disease is the most prominent manifestation of KD (Wang et al., 2011). A large number of studies have shown that in the occurrence of KD, TNF-α, IL-6, TNFR, and other cytokines exist, and these cytokines lead to vascular endothelial cell damage in KD patients, and eventually causing vasculitis (Furukawa et al., 1994; Wang et al., 2011; Kumrah et al., 2020). Tumor necrosis factor (TNF) can bind to TNFR and initiate inflammatory response and physiological functions (Halim et al., 2021). TNFR family is composed of TNFRSF8, OPG, DCR3, etc., and is a growing superfamily with extracellular homologous sequences (Darnay and Aggarwal, 1999; Inoue et al., 2000; Aggarwal, 2003). As an activator of NF-κB receptor, *TNFRSF11A* is also a member of the tumor necrosis factor receptor superfamily (Yang et al., 2019; Glasnovic et al., 2020). Yang et al. (2019) indicated that *RANKL* (a specific ligand of *TNFRSF11A*) *rs2277438* polymorphism increased the risk of rheumatoid disease. Petean et al. (2019) showed that *TNFRSF11A* (*rs3826620*) and its ligand *RANKL* (*rs9594738*) gene polymorphisms were associated with Persistent Apical Periodontitis. Wu et al. (2019) deemed that *TNFRSF11B* (*rs2073617*) gene polymorphism may increase chronic infection with HCV. In a study on osteoporosis, Richards et al. (2009) showed that polymorphism at the *TNFRSF11A* SNP site was significantly associated with fracture risk. Omar et al. (2015) suggested that the rs2073618 gene locus polymorphism of OPG, a key protein downstream of RANK-RANKL signaling pathway, was associated with the development of breast cancer to some degree. These studies suggested that *TNFRSF11A* gene polymorphism may play a role in the occurrence and development of different diseases.

With the development of molecular genetic methods, it is possible to identify susceptibility genes of complex diseases using GWAS and candidate gene methods (Kumrah et al., 2020). Numerous studies have shown that many candidate genes, including FCGR2A (Kuo et al., 2015), ITPKC (Onouchi et al., 2008), VEGF (Ohno et al., 2000), IL-1B (Fu et al., 2019), and ABCC4 (Che et al., 2018a), have been identified as susceptibility genes that increase the risk of KD or coronary complications from KD. However, there is a lack of research on *TNFRSF11A* gene polymorphism and its relationship with KD. In our case-control study, we found that *TNFRSF11A rs7239667C* allele is a protective factor for CAL of KD. To our knowledge, this is the first study to validate the association between *rs7239667 C* allele and KD coronary complications in a population of southern China. We considered that *rs7239667 C* allele may play a significant role in the pathogenesis of CAL of KD.

Although this is the first study to evaluate the association between TNFRS*F11A* gene polymorphism and the risk of KD in children in southern China, there are some limitations that should be noted. First, we focus only on the allele associated with *rs7239667 G* > *C* of *TNFRSF11A*. Polymorphisms at other loci of *TNFRSF11A* gene were ignored. Secondly, due to the nature of the retrospective study design, we only collected cases and controls consistent with race, geography, age and gender, ignoring other factors such as the environment of parents and the presence or absence of infection in eating habits. Third, the sample size of this study is limited and this study lacks functional studies on *TNFRSF11A* (*rs7239667*). Future studies require larger sample sizes to confirm the role of *TNFRSF11A* in susceptibility to KD and relevant functional studies also will be included in our research plan in the follow-up work.

In conclusion, although the current study suggested an association between *TNFRSF11A* gene *rs7239667* and the severity of coronary artery damage in KD. We still need to conduct big data, multi-center studies on the polypeptide properties of *TNFRSF11A* gene to further confirm our current results.

## Data Availability Statement

The original data supporting the relevant research in this article is in the supplementary material. For further inquiries, please contact the corresponding authors of this article directly.

## Ethics Statement

This study was approved by the Medical Ethics Committee of Guangzhou Women and Children’s Medical Center (2014073009). Informed written consent was obtained from the guardians of patients.

## Author Contributions

LZ, KL, YW, and XG designed and organized the study and supervised the whole project. LP, HM, JL, ZJ, and BW contributed to field survey, data collection, laboratory detection, and quality control. DC, LF, YX, and HY performed the data cleansing and statistical analysis. XG, HY, DC, and LZ wrote and critically revised the manuscript. All authors contributed constructively to the editing and drafting of the manuscript and read and approved the final manuscript.

## Conflict of Interest

The authors declare that the research was conducted in the absence of any commercial or financial relationships that could be construed as a potential conflict of interest.

## Publisher’s Note

All claims expressed in this article are solely those of the authors and do not necessarily represent those of their affiliated organizations, or those of the publisher, the editors and the reviewers. Any product that may be evaluated in this article, or claim that may be made by its manufacturer, is not guaranteed or endorsed by the publisher.
